# Flowtaxis of osteoblast migration under fluid shear and the effect of RhoA kinase silencing

**DOI:** 10.1371/journal.pone.0171857

**Published:** 2017-02-15

**Authors:** Brandon D. Riehl, Jeong Soon Lee, Ligyeom Ha, Il Keun Kwon, Jung Yul Lim

**Affiliations:** 1 Department of Mechanical and Materials Engineering, College of Engineering, University of Nebraska-Lincoln, Lincoln, NE, United States of America; 2 The Graduate School of Dentistry, Kyung Hee University, Seoul, Korea; University of California Davis, UNITED STATES

## Abstract

Despite the important role of mechanical signals in bone remodeling, relatively little is known about how fluid shear affects osteoblastic cell migration behavior. Here we demonstrated that MC3T3-E1 osteoblast migration could be activated by physiologically-relevant levels of fluid shear in a shear stress-dependent manner. Interestingly, shear-sensitive osteoblast migration behavior was prominent only during the initial period after the onset of the steady flow (for about 30 min), exhibiting shear stress-dependent migration speed, displacement, arrest coefficient, and motility coefficient. For example, cell speed at 1 min was 0.28, 0.47, 0.51, and 0.84 μm min^-1^ for static, 2, 15, and 25 dyne cm^-2^ shear stress, respectively. Arrest coefficient (measuring how often cells are paused during migration) assessed for the first 30 min was 0.40, 0.26, 0.24, and 0.12 respectively for static, 2, 15, and 25 dyne cm^-2^. After this initial period, osteoblasts under steady flow showed decreased migration capacity and diminished shear stress dependency. Molecular interference of RhoA kinase (ROCK), a regulator of cytoskeletal tension signaling, was found to increase the shear-sensitive window beyond the initial period. Cells with ROCK-shRNA had increased migration in the flow direction and continued shear sensitivity, resulting in greater root mean square displacement at the end of 120 min of measurement. It is notable that the transient osteoblast migration behavior was in sharp contrast to mesenchymal stem cells that exhibited sustained shear sensitivity (as we recently reported, J. R. Soc. Interface. 2015; 12:20141351). The study of fluid shear as a driving force for cell migration, i.e., “flowtaxis”, and investigation of molecular mechanosensors governing such behavior (e.g., ROCK as tested in this study) may provide new and improved insights into the fundamental understanding of cell migration-based homeostasis.

## Introduction

For cell migration studies, considerable emphases have been placed on soluble factor-driven cell migration, i.e., chemotaxis. On the other hand, recent evidences, including our own [1], revealed the importance of fluid flow-induced shear stress in triggering and affecting cell migration, i.e., “flowtaxis”. We showed that mesenchymal stem cell (MSC) migration and its path efficiency are dependent on fluid shear [1]. For bone, recruitment of bone-forming osteoblasts to the bone remodeling site will contribute to bone tissue homeostasis in vivo. The repair of bone fracture and growth of engineered bone tissue may be improved if the osteoblast recruitment (migration) process could be activated and encouraged. It has been established for osteoblasts that fluid shear affects cytoskeletal restructuring, proliferation, differentiation, mechanosensitive signaling, and guidance of bone forming activity [2–8]. Despite these observations that fluid shear regulates osteoblastic cell behaviors, relatively little is known about how osteoblasts migrate in response to fluid shear. Here we subjected osteoblasts to steady fluid flows at physiological level shear stresses and measured cell migration using time lapse imaging and cell tracking software developed in our laboratory. Our results on osteoblast migration revealed a unique short-term sensitivity to fluid shear in contrast to our previous data with MSCs revealing continued shear sensitivity [1]. Further investigation was carried out using molecular silencing of RhoA kinase (ROCK), a regulator of cytoskeletal tension signaling. Interestingly, inhibiting ROCK activated osteoblast migration responses by enhancing cell recruitment to the flow direction, increasing speed, and consequently resulting in an increase in migration length.

Bone is a hierarchical dynamic tissue that experiences significant shear stresses when fluids move through the vasculature, microchannels, and porous regions. Osteocytes embedded in bone lacunae could sense flow-induced mechanical loading and cause bone remodeling cells to respond [9]. Despite osteocytes being the primary mechanosensor in bone, osteoblasts and osteoclasts also respond directly to fluid shear but potentially having differing responses to flow according to their roles in bone remodeling [10,11]. Osteoblasts have been shown to migrate both on solid bone surfaces and through the vasculature to the sites of bone repair guided by osteocytes and chemotaxis signals [12–14]. Shear stress in vessels may also regulate circulating bone progenitor cells in their arrest and extravasation from the vasculature, e.g., intracardially injected MC3T3-E1 osteoblasts could migrate systemically to bone damage sites and contribute to bone remodeling where rat femurs had been agitated with wear particles [15]. Shear stress in bone by interstitial or blood flow is expected to be in the range of about 0.06–30 dyne cm^-2^ [16,17], which will be recapitulated in this study by applying fluid flows with 2, 15, and 25 dyne cm^-2^ shear stresses to determine the potential shear-sensitivity of osteoblast migration. Further, osteoblast migration data will be compared with MSC migration which we obtained at the same shear stress levels [1].

## Materials and methods

### Cell culture

MC3T3-E1 murine osteoblasts (ATCC) were maintained using the growth media composed of alpha minimum essential medium (αMEM) supplemented with 10% fetal bovine serum (FBS) and 1% penicillin/streptomycin. For assessing fluid flow effects on MC3T3-E1 migration, cells were seeded on glass slides (25×75 mm^2^) at 1×10^5^ cells in 1 ml of growth media. Cells were allowed to adhere for 6 h. Then, the media were changed to flow media (serum-reduced media with 5% FBS) and cells were kept overnight. The next day, the cell-cultured slide was assembled with the flow chamber and cells were exposed to steady flows using the flow media. The glass slide was not precoated with extracellular matrix (ECM) protein before cell culture.

### Fluid flow

The FlexFlow fluid flow chamber (Flexcell International) was assembled as directed by the manufacturer (Fig A in S1 File, also see S1 Fig from our previous publication [1]). The flow route consisted of Masterflex L/S 16 tubing that connected a media reservoir, peristaltic pump, Osci-Flow flow controller, pulse dampeners, and the flow chamber mounted on the fluorescent microscope. The flow regimens could be controlled by the peristaltic pump and the Osci-Flow device which were governed by StreamSoft v. 4.1 software provided by the company. The volume flow rate to achieve the desired shear stress level was determined from the chamber dimension, tube size, and the media viscosity. The shear stress applied to the cells was assumed to be the wall shear stress for flows between two parallel plates with infinite depth assuming the 2D Newtonian flow. More details of the flow device and shear stress calculation are reported in our previous article [1].

The flow system was sterilized by flowing 70% ethanol for 10 min through the tubing, which was then flushed out twice with deionized water. For tests, the reservoir was filled with 400 ml of serum-reduced flow media and kept in a 37°C water bath. The media were then circulated until all air bubbles were removed from the tubing. For each test, the cell-seeded glass slide was assembled to the FlexFlow device using a negative vacuum pressure and the flow media were primed for 30 sec to remove bubbles that could be introduced during the slide changing process. After inspection of the flow lines and vacuum seal, the flow chamber was placed on an inverted microscope (Leica DMI4000). A steady flow was applied for 120 min at 2, 15, or 25 dyne cm^-2^ shear stresses (labeled as FF2, FF15, and FF25, respectively). The static unflowed control was used with the same flow device setup but not exposed to flow.

### Time lapse imaging and data processing

Time lapse image stacks of the cells were obtained by recording the phase contrast images once per minute during the flow with the inverted microscope. A region of the slide away from the edge and containing many free cells was chosen for imaging. Cells that touched other cells, exited the frame, or were washed away by the flow were excluded in the post-analysis. The full details of image processing and cell migration data analysis followed our published protocols [1]. Briefly, obtained images were first corrected to deal with potential microscope and device drifts. This was achieved by using the template matching plugin of the open-source FIJI software [18]. Then, image segmentation and automated cell outline tracking was performed using the time lapse analyzer (TLA) [19]. Segmentation of phase contrast images was performed by combining two binary masks, the first from Otsu thresholding of the image entropy and the second created using Sobel edge detection. Detected cell outlines were used to determine the cellular centroid position at each time frame. By connecting centroid points, raw cell migration tracks for each cell were obtained. Cell migration tracks and binary mask videos were exported to the Matlab script developed in our lab to further quantify cell migration. These include cell migration plots (raw migration and compass plots), percentage of cells and time migrating with/against the flow, migration speed at each time frame, displacement length, confinement ratio, arrest coefficient, etc. As a measure of collective cell migration trend, the group dispersion was calculated at each time frame by using the root mean square (RMS) displacement (*X*_*RMS*_):

$${X(t)}_{RMS}=\sqrt{\frac{1}{N}\sum_{i=1}^{N}{\left[x_{i}(t)-x_{i}(0)\right]}^{2}}$$ (1)

where *t* is time, *N* is the total number of measured cells, and *x*_*i*_ is the cell position vector for the *i*-th cell. The term *x*_*i*_(*t*) − *x*_*i*_(0) is the displacement of *i*-th cell at time *t* from the initial starting position *x*_*i*_(0). This calculation method produces an ensemble average, the average displacement of all the cells in the measured group at each time point. The motility coefficient, the slope of the plot of RMS displacement vs. square root of time which indicates the strength of cell mobility analogous to the diffusion coefficient [1,20], was also calculated. Definitions of other parameters are described in the Results sections.

### Silencing ROCK via small hairpin RNA (shRNA)

To reveal the role of ROCK in osteoblast migration under fluid flow, MC3T3-E1 cells with stable knockdown of ROCK were established. For this, shRNA-ROCK1 plasmid (Santa Cruz Biotechnology, sc-36432-SH) and lipofectamine 2000 transfection reagent were used as described in our previous publications [1,21]. Briefly, after 24 h transfection with shRNA plasmid, the cells were washed with phosphate buffered saline and placed in selection media containing 2 μg ml^-1^ puromycin. Cells with silenced ROCK were then selected from puromycin resistant cells, which were then established as a stably silenced cell line. This procedure was repeated to create the vector control using the green fluorescent protein (GFP)-tagged control plasmid (Santa Cruz Biotechnology, sc-108083). All the cell migration tests were completed with using the vector control cells unless noted as ROCK-shRNA (ROCK-sh) in the figure. Stable knockdown of ROCK by shRNA was confirmed by western immunoblotting after subcultures in comparison with the GFP vector control (Fig C in S1 File). The effect of fluid shear was compared for ROCK-silenced cells and vector control at a representative shear stress, 25 dyne cm^-2^. We note that using the GFP plasmid vector control may have a slight limitation in that the comparison is between a shRNA sequence that limits ROCK mRNA and a sequence that produces GFP, an exogenous protein. The use of a scrambled-shRNA vector control would have been the best control sample, as there might be unintended effects from shRNA manipulations.

### Statistics

One way analysis of variance (ANOVA) with a Tukey-Kramer post-hoc test was used to assess statistical significance. The data were checked to ensure that the ANOVA assumptions were met. If necessary for ANOVA assumptions to be met, skewed data was log_10_ transformed before applying statistical methods and back-transformed to present the results (Table A in S1 File). The data are presented as mean ± standard error of measurement (SEM) and statistical significance is noted with symbols in each figure.

## Results

**Fig 1:**
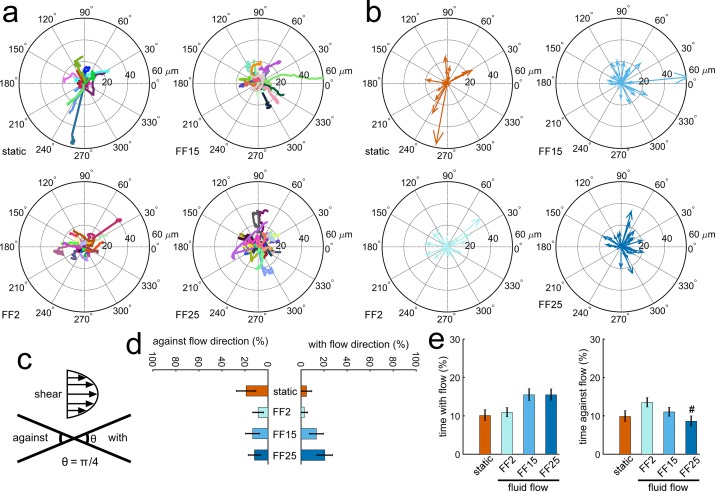
Fluid shear effects on osteoblastic cell migration could be detected. MC3T3-E1 osteoblasts had a trend of migrating with the flow direction with increasing shear and spent less time migrating against the flow, especially under high shear. (a) Individual cell raw migration tracks. Flow was given from left to right for fluid flow (FF) cases. FF2, FF15, and FF25 denote fluid shear stress of 2, 15, and 25 dyne cm^-2^, respectively. (b) Compass plots connecting starting and ending positions. (c) Cell migration is considered to be with the direction of flow if the migration angle in the compass plot is within ± π/8 of the flow direction. (d) Cells showed an increasing trend of migrating with the flow with increasing shear. The percent of cells migrating against the flow did not have a clear trend. (e) The cells under higher shear (FF15, FF25) tended to spend more time migrating with the flow. The FF25 group spent the least time migrating against the flow (#: p < 0.05 compared with FF2).

MC3T3-E1 osteoblastic cell raw migration tracks are shown in Fig 1A, in which each cell track is distinguished by color and the track initiation is shifted to the center of the plot. For fluid flow (FF) cases, flow was applied from left to right. The image segmentation and exclusion criteria were checked to ensure only individual cells with good segmentation were included. Compass plots (Fig 1B) were drawn by connecting the cell migration track from beginning to the ending position. The percentages of cells and time migrating with and against the flow direction were quantified for the entire 120 min test period. A cell was considered to migrate with the flow if the terminal track in the compass plot was within ± π/8 of the flow direction, and migration opposite to this was counted to be against the flow (Fig 1C). There was an increasing trend of osteoblastic cell number recruited to migrate with the flow direction with increasing shear stress (Fig 1D) but not reaching statistical significance. Cells subjected to 15 and 25 dyne cm^-2^ spent more time migrating with the flow (Fig 1E), but again data did not reach statistical significance. The cells subjected to 25 dyne cm^-2^ spent the least time migrating against the flow especially when compared with FF2.

**Fig 2:**
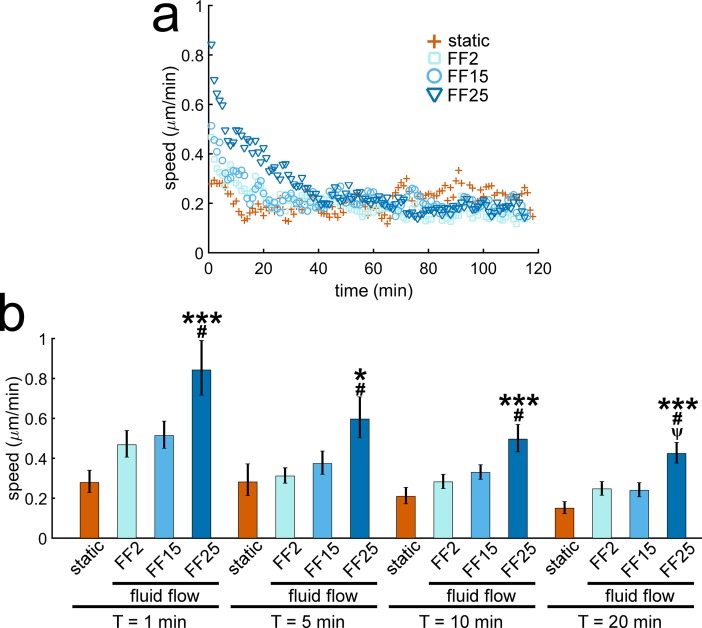
Osteoblast migration speed shows shear stress dependence but only for a short period after the onset of the flow. (a) MC3T3-E1 osteoblasts under fluid flows showed peak migration speeds after the flow onset, which then decreased. The flowed cells initially migrated faster than the static control, but after about 30 min there were no notable differences among test groups. (b) FF25 showed significantly greater cell migration speed at 1, 5, 10, and 20 min. * and ***: p < 0.05 and 0.001 compared with static control; #: p < 0.05 with FF2; ψ: p < 0.05 with FF15.

Cell migration speed quantified for each time frame is shown in Fig 2A. It is an average speed of all cells observed at each minute of measurement (shown without error bars for clarity; see Fig B in S1 File with error bars). MC3T3-E1 osteoblasts showed peak migration speed with the onset of the flow and had a decreasing trend thereafter. The peak speed increased with increasing shear stress (e.g., 0.28, 0.47, 0.51, and 0.84 μm min^-1^ for static, FF2, FF15, and FF25, respectively, at 1 min, Fig 2B). Cell migration speeds in the flowed groups were greater than static control for up to about 30 min after the flow onset. FF25 displayed the fastest speeds during this initial period, which were significantly greater than the static control and other FF cases (Fig 2B). After about 30 min, there were no significant differences in osteoblast migration speeds among test groups. The migration speed data indicate that osteoblasts may have fluid shear sensitivity but only for a short period of time after the flow onset.

**Fig 3:**
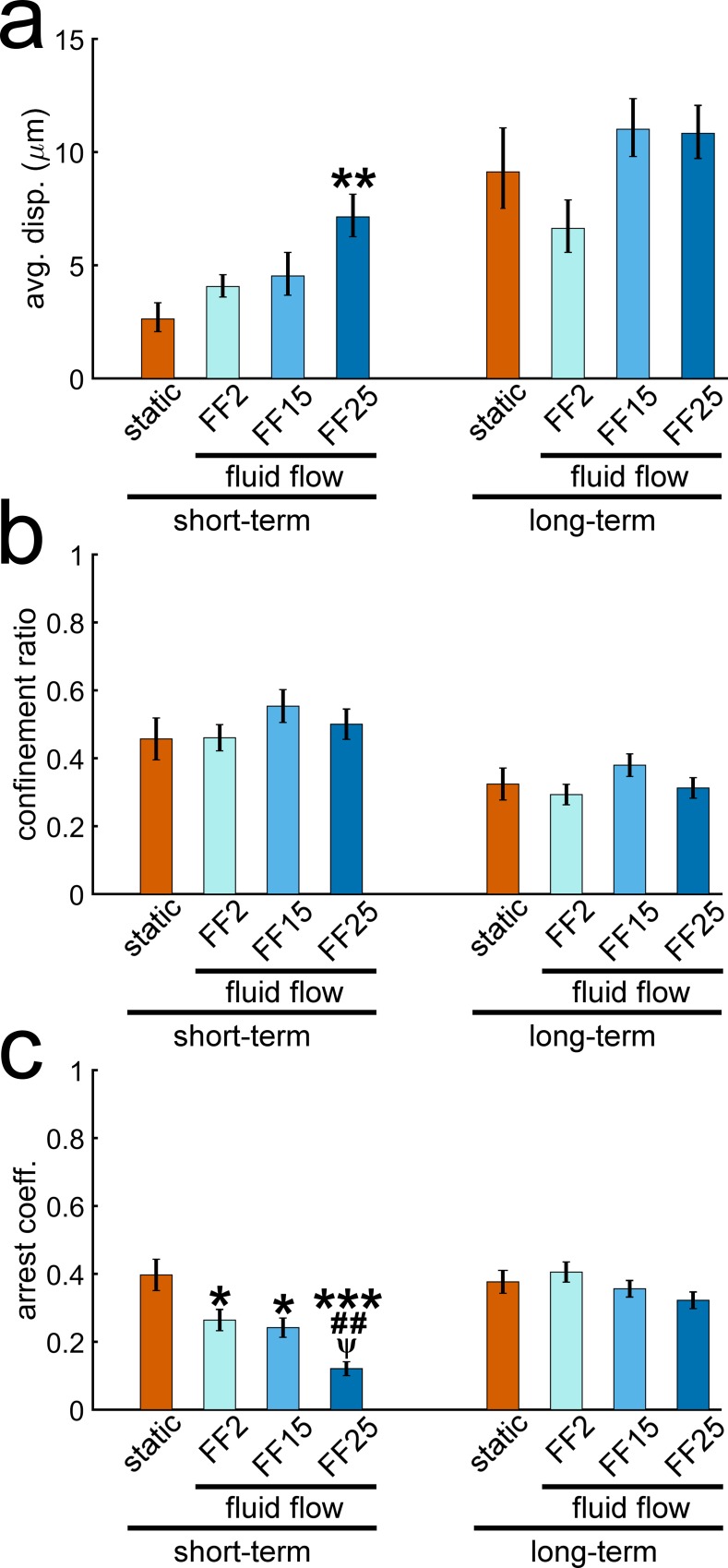
The displacement length and arrest coefficient of osteoblast migration show fluid shear sensitivity but only for a short period after the flow onset. Data were presented for the short-term (from 0 to 30 min) and long-term (the entire tracking from 0 to 120 min) durations. (a) The short-term displacement increased with shear. FF25 migrated significantly further than the static control. These differences were not observed in the long-term data. (b) There was no significant difference in the confinement ratio (directness of the migration path) with respect to shear stress. The ratio generally decreased for all test conditions as time increased, indicating reduced path efficiency with time. (c) Flow groups had significantly smaller arrest coefficients (less time paused) compared with the static control in the short-term data, which was not observed in the long-term result. *, **, and ***: p < 0.05, 0.01, and 0.001 compared with static control; ##: p < 0.01 with FF2; ψ: p < 0.05 with FF15.

Data in Figs 1 and 2 may suggest distinction between short and long-term effects of fluid shear on osteoblast migration. In Fig 3, cell migration behaviors quantified for the short-term (up to 30 min) and long-term (entire 120 min) periods are shown. The cell displacement length for the short-term period was found to depend on the shear stress level, with FF25 having the greatest displacement and reaching statistical significance compared with the static control (Fig 3A). This fluid shear sensitivity in displacement did not continue throughout the entire period as seen in the long-term result. The confinement ratio (Fig 3B), a measure of the directness of a cell migration path, was calculated by dividing the displacement by the total path length (efficient straight path approaching 1 and tortuous path 0). The short-term confinement ratios were generally higher than the long-term ratios, implying that cell migration paths were initially more direct and then became less direct as the time increased. However, there were no marked differences in the confinement ratio with respect to shear stress either for short or long-term periods. The arrest coefficient (Fig 3C) measures the percent of time that a cell is paused during migration. A cell was considered paused if the cell speed was less than one standard deviation below the average cell speed of the static condition. The static average speed was 0.21 μm min^-1^ with a standard deviation of 0.05 μm min^-1^, which yielded an arrest threshold of 0.16 μm min^-1^. The arrest coefficient exhibited strong short-term shear stress dependence. Cells in all flowed groups for the short-term spent significantly less time paused compared with the static control. Specifically, FF25 spent significantly less time paused relative to all other conditions. On the other hand, again, the differences in short-term arrest coefficients were not detected in the long-term data. Combined results indicate that osteoblast migration may have fluid shear sensitivity but with limited time duration after the flow onset.

**Fig 4:**
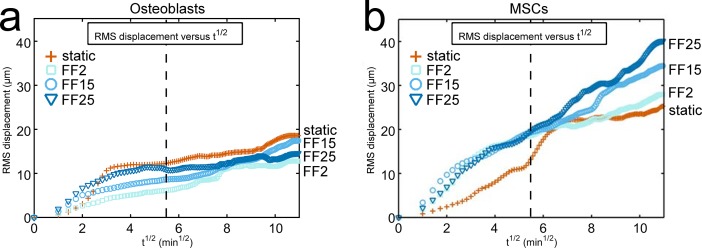
RMS displacement plots show differences in osteoblast vs. MSC migration under fluid shear. (a) The RMS displacement of all participating cells, a holistic measure of migration tendency, was plotted for osteoblasts. (b) Data for MSCs were also included for comparison (reprinted from our publication [1] with permission from the Royal Society, see S2 Fig). Note that the same flow conditions to produce the same shear stresses were applied for both cells. MSCs showed continued fluid shear effect to induce migration. MSCs also showed prolonged shear stress dependency, e.g., the RMS displacement at 120 min was in the order of static < FF2 < FF15 < FF25. These trends lacked in the MC3T3-E1 osteoblast migration. In the RMS displacement plot for osteoblasts, shear stress dependency was only visible for a short period of time after the flow onset. The capacity of fluid shear to induce cell migration was reduced for osteoblasts with time. The dashed vertical line marks 30 min after the flow onset.

The RMS displacement may be a more holistic measure of cell migration tendency, as was used in our study on MSCs [1]. It calculates an average-sense migration length for all the cells observed (Eq 1), and consequently reflects all combined characteristics of cell migration including cell speed, displacement, confinement ratio, and arrest coefficient. The RMS displacement plots for MC3T3-E1 osteoblasts are presented in Fig 4A. For comparison, MSC data under the same fluid shear stresses are included in Fig 4B (from our publication [1], see S2 Fig). For osteoblasts, detectable fluid shear dependency in the RMS displacement plot could only be seen for a very short time period after the flow. This is reminiscent of the cell speed data in Fig 2. A dashed line is drawn in the RMS displacement plot to mark 30 min after the flow onset. For FF25, an initial fluid shear adaptation was followed by a long plateau. Both FF2 and FF15 had a more gradual shear adaptation with less of a plateau. Potentially due to the lack of long-term shear dependency, at the end of 120 min osteoblasts under static condition showed even greater RMS displacement relative to flowed cells. These osteoblast migration behaviors are in sharp contrast to MSC migration. When exposed to the same fluid shear stresses, MSCs migrated continuously with little plateaus and generally showed greater RMS displacements compared with osteoblast counterparts, suggesting that MSCs under fluid shear may be more mobile than osteoblasts. Further, MSCs displayed a sustained trend of increased migration with increasing shear, e.g., RMS displacement at the end of 120 min was in the order of static < FF2 < FF15 < FF25, which was not the case for osteoblasts. The RMS displacement plots obtained under the same flow conditions thus illustrate the potential differences of osteoblast vs. MSC migration with respect to sustainable fluid flow effect and sensitivity to shear stress magnitude.

Differences in osteoblast vs. MSC migration may further be revealed by the motility coefficient. In the RMS displacement plot against the square root of time, as noted above, the slope is defined as the motility coefficient and has a physical meaning analogous to the diffusion coefficient (or diffusivity) of the Fick’s first law of diffusion [1,20]. As in Table 1, the motility coefficient of MC3T3-E1 osteoblast migration showed shear stress dependency (FF2 < FF15 < FF25) but only for short periods of time after the flow onset (e.g., up to 30 min). Moreover, osteoblast static control had even greater motility coefficients than flowed osteoblasts in several time frames. For MSCs, motility coefficients were generally higher than those of osteoblasts especially for FF cases, again indicating that MSCs may be more mobile than osteoblasts under flows. Unlike osteoblasts, flowed MSCs had motility coefficients greater than static control for both short and long-terms (the only exception was FF2 and static comparison at 120 min). A strong short-term MSC migration induction at specific shear stress level (FF15), as we reported [1], can also be seen by the higher motility coefficient for MSCs under FF15 at 5 min. When assessed for the entire flow period (120 min), MSCs displayed an increasing motility coefficient with shear stress (FF2 < FF15 < FF25), suggesting a prolonged fluid shear sensitivity acting on MSCs.

**Table 1:** Motility coefficient obtained as a slope of the RMS displacement vs. t^1/2^ plot.

	Osteoblasts					MSCs				
	Short-term				Long-term	Short-term				Long-term
	5 min	10 min	20 min	30 min	120 min	5 min	10 min	20 min	30 min	120 min
**Static**	1.83	3.93	3.65	2.62	1.29	1.30	1.71	2.37	2.67	2.54*
**FF2**	1.35	1.43	1.29	1.13	1.22	3.51	4.74	4.48	3.68	2.00*
**FF15**	2.57	2.26	1.77	1.42	1.40	4.99	4.54	3.61	3.26	2.87*
**FF25**	3.43	3.20	2.59	1.95	0.80	3.71	4.33	4.25	3.65	3.55*

* Data represented from our previous publication [1] with permission from the Royal Society.

**Fig 5:**
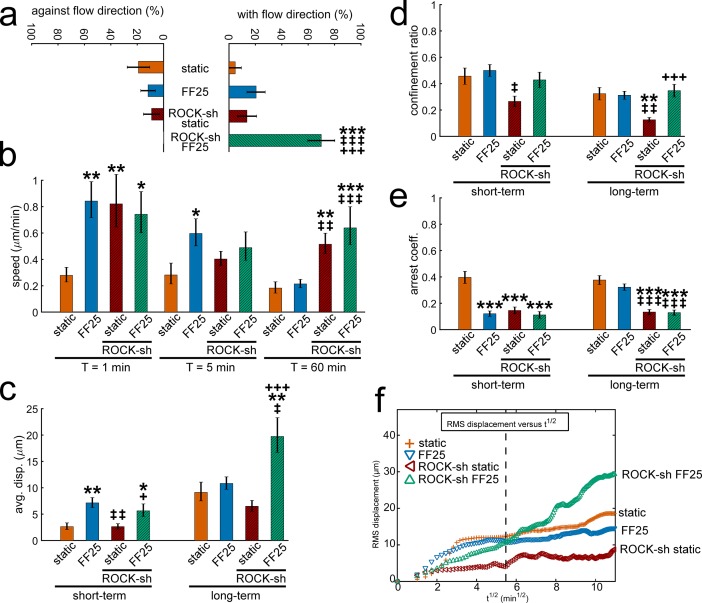
Osteoblast recruitment in the flow direction and motility under shear are increased with ROCK interference. (a) Osteoblasts with ROCK-shRNA (ROCK-sh) under FF25 showed significantly greater number of cells migrating with the flow direction. (b) Even after the initial period, the speeds of ROCK-silenced cells were greater for both static and sheared conditions (e.g., 60 min). (c) The displacement of ROCK-shRNA FF25 group assessed after 120 min was significantly greater compared with other conditions. (d) The ROCK-shRNA static group was less confined in migration path. (e) The ROCK-shRNA cells paused significantly less during the migration. (f) The RMS displacement shows that the collective migration of the ROCK-shRNA FF25 group was continued throughout the measurement time resulting in greater RMS displacement at 120 min. The dashed vertical line marks 30 min after the flow onset. *: comparison with vector control static. ‡: comparison with vector control FF25. +: comparison with ROCK-shRNA static. Single, double, and triple symbols represent p < 0.05, 0.01, and 0.001, respectively.

To gain some insight into the molecular mechanism controlling osteoblast migration, we repeated the test under interference of ROCK, one of the key cytoskeletal mechanosensors (see stable silencing in Fig C in S1 File). In the raw migration tracks and compass plots for MC3T3-E1 osteoblasts under ROCK-shRNA (Fig D in S1 File), significantly increased migration with the flow direction under ROCK-shRNA is noticeable. This can be seen as percent cells (Fig 5A) and time (Fig E in S1 File) with the flow. Cells with silenced ROCK could also migrate at higher speeds even after the initial period for both static and FF25 cases, e.g., 60 min speed data in Fig 5B (see full set of data with times in Fig F in S1 File). Increased motility under ROCK silencing resulted in significantly increased displacement under sheared condition (ROCK-shRNA under FF25, Fig 5C). On the other hand, cells with ROCK-shRNA under static condition had relatively less displacement regardless of higher speed, which can be attributed to significantly decreased confinement ratio (Fig 5D) such that ROCK-silenced cells without flow mostly wandered with no directional persistence. Increased motility by ROCK-shRNA was also evident in the arrest coefficient (Fig 5E), showing that ROCK-silenced cells had significantly less stops during migration. As in the RMS displacement plot (Fig 5F), cells with ROCK-shRNA had continued migration tendency especially under fluid shear. The ROCK-shRNA FF25 group showed greater RMS displacement due to increased persistent migration at a higher speed with greater path efficiency. Distinction between short-term and long-term flow sensitivities for osteoblasts (as described earlier) disappeared with ROCK interference. The motility coefficient also indicates the existence of long-term shear dependency under ROCK-shRNA (Table B in S1 File, 120 min). The ROCK-shRNA static group lacked a directed migration as noted with confinement ratio, which resulted in relatively lower RMS displacement and motility coefficient.

## Discussion

Migration and recruitment of osteoblasts to the bone remodeling site is important for fracture repair, engraftment of prosthetics, and of general interest for developing treatments for bone diseases such as osteoporosis. Osteoblast migration/recruitment is known to be activated by various biochemical factors [14,22,23]. Our results demonstrate that osteoblast migration can also be affected by fluid flow-induced shear stress to which the cells in bone are exposed. Under physiologically relevant level shear stresses, recruitment of osteoblasts to participate in the migration with the flow and the time spent with the flow direction had increasing trends with increasing fluid shear. Furthermore, cell migration speed, displacement length, arrest coefficient, and motility coefficient displayed notable changes depending on shear stress magnitudes. Interestingly, such osteoblastic fluid shear sensitivities were found to be vital only for a short period of time after the flow onset, which became less organized after the initial activation. As a result, osteoblast migration behaviors under flow were quite different from those of MSCs that showed prolonged migration stimulatory effect by flow and well-maintained shear stress sensitivity.

Cells in bone are sensitive to the time parameters of loading including the frequency, rest period, and shear rate [24–27]. It is recognized that oscillatory fluid flow in bone is ubiquitous, which is caused by the loading and unloading of bone that drives interstitial flows through microchannels. Many studies have adopted oscillatory flows to test the fluid shear control of bone cell behaviors [3,5,8,11]. Regardless of the biomimetic nature of oscillatory flows, on the other hand, steady laminar flows have also been utilized in many studies. These studies exploiting steady flows also aimed to examine the mechanotransduction of bone cells. Moreover, many bone tissue engineering approaches have adopted steady flows in the flow bioreactors. It was evidenced that steady and oscillatory flows may differentially regulate bone cell behaviors such as actin stress fiber development, intracellular signaling, and differentiation [2,5,11]. In some cases, interestingly, oscillatory flow showed less stimulatory effect than either steady or pulsed flow [28]. Our data obtained with steady flows at physiologically relevant levels of shear stress allowed for the identification of the initial shear stress-sensitive window for the osteoblast migration. We showed that most stress-sensitive adaptations in osteoblast migration occurred during this initial period, after which the effects decreased. Such a decrease in osteoblast migration sensitivity to steady flow may be in analogy with reported results showing decreases in osteoblastic differentiation and remodeling after being exposed to continuous flows [25]. It is acknowledged that results of our study with steady flow may not be directly applied to in vivo osteoblast migration in which oscillating shear may play an important role. However, our data can be used for comparison with other studies adopting steady flows for osteoblastic cell gene expression, proliferation, and differentiation as mentioned above. Our setup could also reveal the potential difference in the flowtaxis of osteoblasts vs. MSCs under the same steady flows, as highlighted in Fig 4.

Taken together, a contextual framework can be provided here to facilitate interpretation of the results obtained with steady flow. Steady flow has been useful for studying fundamental responses of bone cells, such as secretion of tissue remodeling factors and the adaptation of cell morphological features and signaling cascades. A more useful context would be that of tissue engineering with perfusion flow acting both as a mechanical stimulus for cell inoculation and as a way of nutrient transport. For instance, how to induce cells to penetrate inside the scaffold within the flow bioreactors can be studied with steady flows. Several data support these rationale for steady flows. At the individual cell level, unidirectional fluid flow could promote cytoskeletal remodeling [4]. An increase in mechanical resistance to steady flow by upregulating cytoskeletal crosslinking resulted in further maturation of bone tissues [11]. Starting with a tissue scaffold and seeding with stem cells, it was found that steady fluid flow effectively guided osteogenic lineage commitment even without soluble factors [27]. After cells were committed to an osteogenic lineage, their bone-forming activities could be controlled by unidirectional shear with increasing or decreasing stepwise magnitude, which respectively increased or decreased scaffold mineralization and homeostasis-regulating soluble factors [26]. Within this context, our results may help address the knowledge gap in osteoblast migration in mechanically active milieus. Our results provide useful baseline data, considering few studies have measured osteoblast flowtaxis either in controlled steady or oscillatory shear environments.

Our results showed that osteoblast migration under steady flow may only have short-term shear dependency. It is possible that shear sensitivity in osteoblast migration may be restored with the insertion of rest periods. As an example (but not targeting migration), the presence of rest periods improved the response of bone cells to flow in mechanotransduction, differentiation, and remodeling activity [25,27]. While our study focused on the role of shear stress magnitude, bone cells are also sensitive to the other shear parameter, e.g., rate of shear [24]. A future study may be designed to more effectively incorporate time variance and initial stress kicks, which would be useful for improved understanding of osteoblast migration and recruitment and resultant bone tissue homeostasis.

Data comparisons between osteoblasts and MSCs lead to a tentative conclusion that osteoblasts have less sustainable fluid shear sensitivity than MSCs under the same steady flow conditions. The changes in migration behavior among cell types could be due to intrinsic differences in the rate of cytoskeleton and focal adhesion remodeling. It is established that focal adhesion turnover and related cytoskeletal remodeling are essential processes in cell adaptation to mechanical stimulation, and the rate of such remodeling processes could differ among cell types and by the mode of stimulation. For instance, for sheared endothelial cells [29], initial focal adhesion remodeling started within 2 min of flow shear onset, focal adhesion kinase (FAK) was recruited to the leading cell migration edge within 30 min, and the cell had polarized migration with marked cytoskeletal changes in 60 min. Studies comparing focal adhesion and cytoskeletal remodeling rates for osteoblasts and MSCs under flow may help reveal the causes of observed differences in migration. Practically, once the cell-specific focal adhesion-cytoskeletal remodeling timeline is known, the fluid flow stimulation may be tailored for tissue engineering purposes to most effectively achieve the desired results of cell migration/penetration into the bone tissue engineering scaffold.

Our data evidence that osteoblasts may have a transitory migration response with clear short-term migration trends and long-term behaviors that may be indicative of altered temporal processing. MC3T3-E1 osteoblasts had a narrower time window of shear stress sensitivity and had less persistent migration compared with MSCs. For MSCs, improved migration path efficiency, more persistent migration, and more cells participating in migration resulted in higher motility coefficients for both short and long-term flow conditions relative to osteoblasts. Since bone has a natural, well-organized network of sensing and remodeling cells, it might not be critically requested to have continuous cell migration within the bone (relative to stem cells which may tend to keep migrating, e.g., through the vasculature). Instead, in the bone tissue a preference may be given to the nearby osteoblasts to migrate over relatively short distances to the remodeling site. Additionally, MSCs are more likely to experience unidirectional fluid flow in vivo, whereas steady flow is not a characteristic physiological stimulus for osteoblasts which typically experience oscillatory flow. More flowtaxis studies will be needed that explore the role of oscillatory and intermittent shears in controlling osteoblast migration, which could be useful for understanding the nature of bone cell migration and resultant bone tissue homeostasis.

The small GTPases of the Rho family, including ROCK, can control actin stress filament remodeling. Inhibition of ROCK is currently explored to improve cell transplantation in stress-sensitive cells, to control cell attachment to unfavorable surfaces, and to increase cell migration in static environments [30–34]. The increased cell motility response in static culture by ROCK inhibition is proposed to act through modified actomyosin regulation [35]. It is likely that ROCK interference may also allow under a flow shear environment higher cytoskeletal deformation and reduces the stimulus level required for remodeling, resulting in increased cell migration. Our data supports the premise that ROCK inhibition in osteoblasts increases motility and extends the result to include fluid shear environments, e.g., ROCK-shRNA significantly increased osteoblast recruitment in the flow direction and extended the shear-sensitive time window. These results are in line with our previous finding that ROCK inhibition increased MSC migration under flow [1]. Such information could be useful for targeted tissue engineering approaches that require long-distance migration of bone forming cells.

Potential mechanisms may also include competition between ROCK and Rac. Since ROCK and Rac pathways are known to have an antagonistic relationship, silencing ROCK should follow with an increase in Rac. Rac can localize to the leading edge of the cell migration and in some cases may drive the membrane protrusion behavior [36]. Thus, ROCK interference is expected to increase cell migration under flow via upregulating Rac-mediated lamellipodia formation at the leading edge of the cell migration. A future study can target the role of ROCK-Rac crosstalk in osteoblast flowtaxis. Other molecular mechanosensors may also play a role. Of particular interest is cadherin cell-cell adherens junction. The actin binding cadherin cell-cell junction may facilitate communication of mechanical signals among cells when the cells are collectively migrating (migration while keeping cell-cell contacts) [37]. This would provide advanced knowledge on the ensemble migration of osteoblasts (bone-lining cells) in vivo, which may suggest a useful strategy for improving osteoblast engraftment with bone tissue engineering scaffold in dynamic bone remodeling environments.

Our previous work suggested that seeding osteoblastic cells on ECM protein-uncoated glass slides causes α_V_β_3_ integrin to be the main cell attachment site [38]. This was achieved through transmembrane integrin α_V_β_3_ binding to vitronectin adsorbed on the slide from serum proteins. While vitronectin can be found in the connective tissue and is part of the disease repair process [39], a more bone-mimicking ECM milieu, e.g., type-I collagen coating, may provide more physiologically relevant osteoblast migration data. Another substrate consideration would be topographic modification in both micro and nanoscale, since bone cells in vivo are exposed to such topographies. Our experience of surface topographic modifications and their effects on osteoblastic cell behavior [40,41] could be used to explore the substrate topographic control of osteoblast migration under fluid shear, as we evidenced for MSC sensitivity in cytosolic calcium response under nanotopography-flow co-exposure [42].

It is not unreasonable to extrapolate that fluid shear would be a vital factor in vivo for osteoblast recruitment for bone tissue homeostasis. Our results demonstrate that fluid shear can directly activate bone cell migration, although the stimulatory effects were mostly within short time periods. The fluid shear activation of osteoblast migration can definitely work in tandem with chemotaxis such as soluble factors secreted by other bone cells (osteocytes, osteoclasts), oxygen gradient, etc. Since the shear ranges tested in this study are known to activate bone remodeling responses in primordial cells related to proliferation or differentiation, our study of bone forming osteoblast migration/recruitment under fluid shear may be of significance as a first step to understand mechanical bone remodeling. Further probing molecular mechanisms, as partly achieved through ROCK silencing studies, related to heterogeneous osteoblast migration responses to shear (e.g., why short-term shear sensitivity phases out with continuous flow) may provide advanced knowledge on the fluid shear control of bone remodeling and regeneration and for functional bone tissue engineering.

## Conclusions

Our study provides new and possibly unexpected insights into osteoblastic cell migration. The results demonstrated that steady fluid shear can initiate MC3T3-E1 osteoblastic cell migration in a stress magnitude-dependent manner. However, the fluid shear sensitivity was substantial mostly for the initial time span. As a result, a short-term shear-sensitive window, e.g., up to about 30 min, was identified during which osteoblast migration behaviors such as speed, displacement, arrest coefficient, and motility coefficient were dependent on the shear stress level. Osteoblasts had reduced shear sensitivity after this window and demonstrated a decrease in migration ability. Osteoblastic cell migration was enhanced with interference of ROCK, which suggested that less cytoskeletal resistance to remodeling may support cell migration under flow. The short-term shear sensitivity of osteoblast migration formed sharp contrast to MSC migration which had a more robust migration response and sustained shear sensitivity. Future investigations of osteoblast flowtaxis with further considerations on the effect of flow and time regimens, ECM coating and substrate topography modification, and in-depth molecular mechanisms may lead to better understanding of bone homeostasis in vivo and improved bone tissue engineering protocols.

## Supporting information

S1 Fig This is the figure we reprinted as Fig A in S1 File from our previous publication [1] with permission from the Royal Society. (PNG)

S2 Fig This is the figure we reprinted as Fig 4B from our previous publication [1] with permission from the Royal Society. (PNG)

S1 File This supplementary information file contains the fluid flow device, statistics, average cell speed plot with error bars, and migration data under ROCK-shRNA. (PDF)
