# Supplementary figures and images for: Flowtaxis of osteoblast migration under fluid shear and the effect of RhoA kinase silencing
Source: PLoS One. 2017 Feb 15;12(2):e0171857. doi: 10.1371/journal.pone.0171857 (PMC5310897; doi:10.1371/journal.pone.0171857)

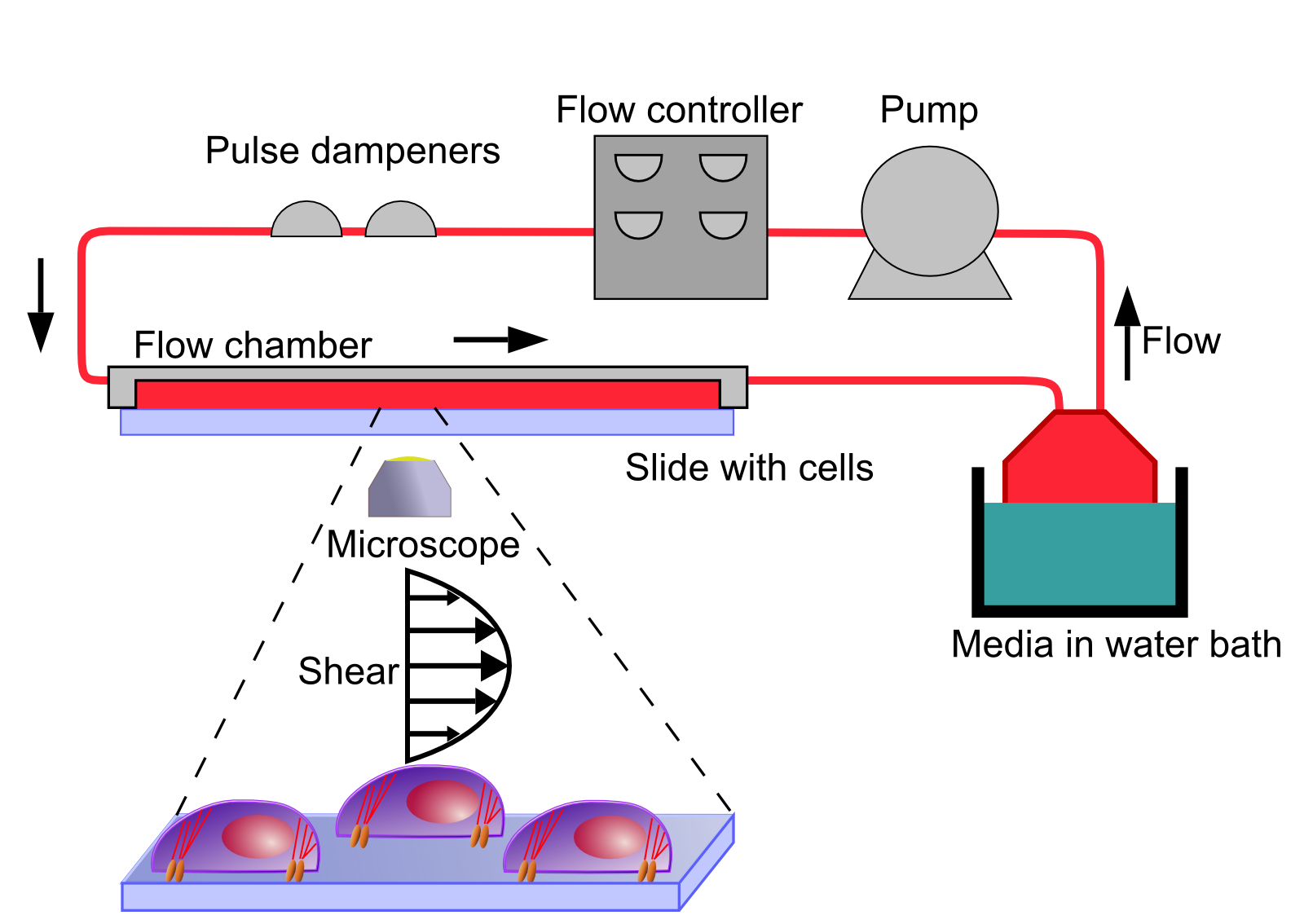

Supplement: S1 Fig — (PNG) [file pone.0171857.s001.png]

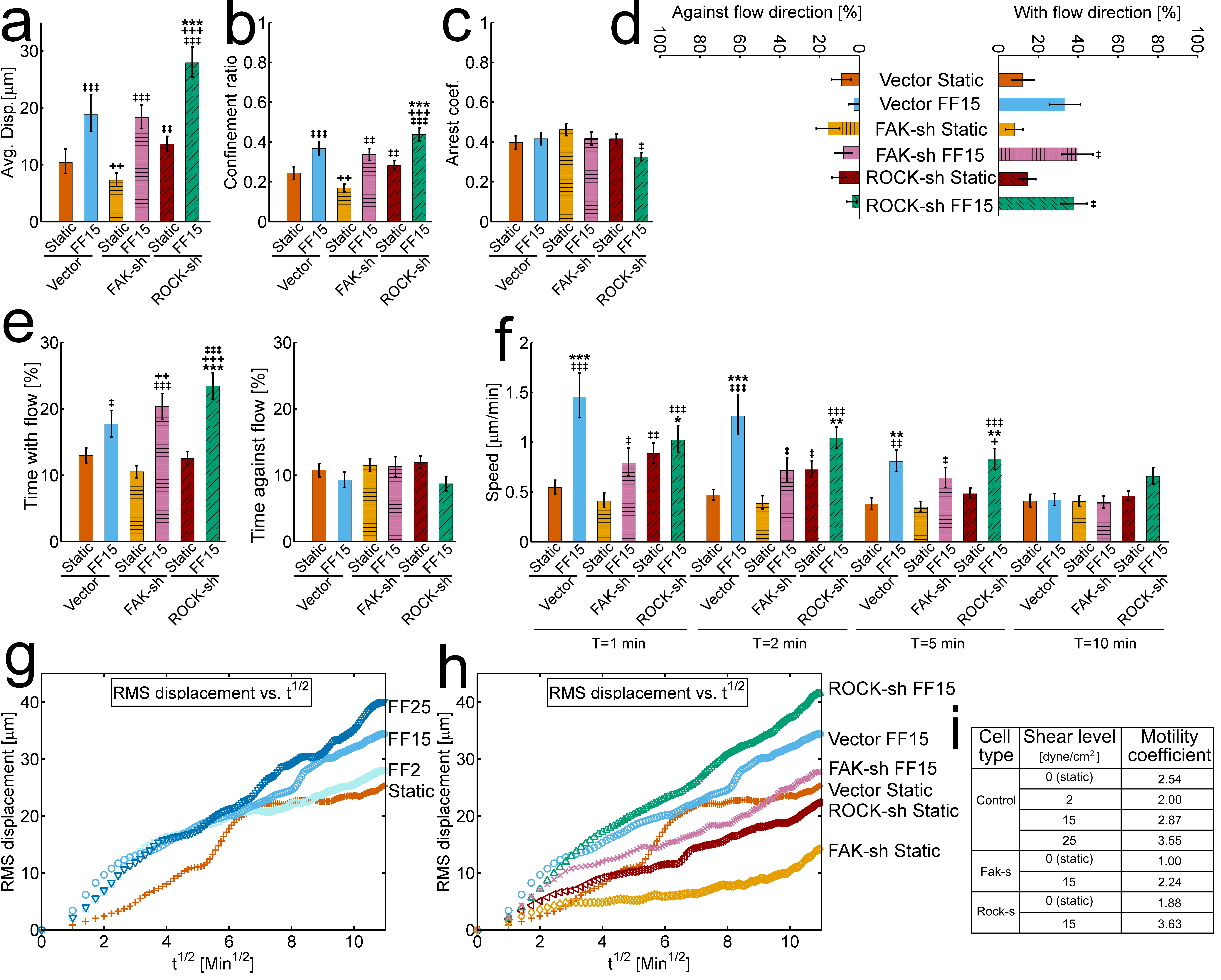

Supplement: S2 Fig — (PNG) [file pone.0171857.s002.png]
