# Supplementary material for: Flowtaxis of osteoblast migration under fluid shear and the effect of RhoA kinase silencing
Source: PLoS One. 2017 Feb 15;12(2):e0171857. doi: 10.1371/journal.pone.0171857 (PMC5310897; doi:10.1371/journal.pone.0171857)
Supplement: S1 File — (PDF) [file pone.0171857.s003.pdf]

SUPPLEMENTARY INFORMATION

Fluid Flow Device

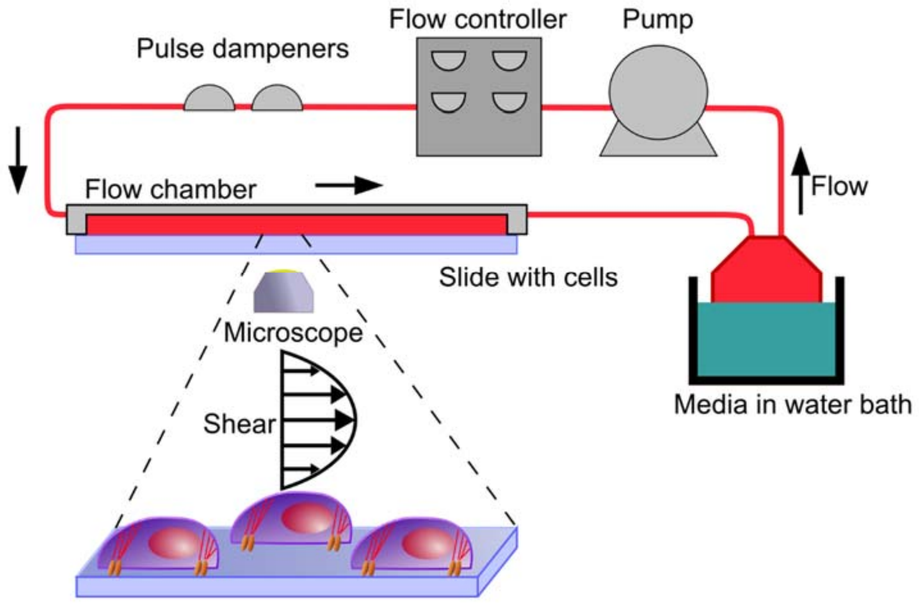

Fig. A. Schematic of fluid flow device. Figure is reprinted from our previous publication [1] with permission from the Royal Society.

Statistics

A minimum of 21 cells were quantified for the static condition and a minimum of 30 cells were quantified for each fluid flow conditions. For ROCK-shRNA, no less than 20 cells were quantified for each condition. Data transforms were applied to meet the assumptions of ANOVA if the ANOVA residuals were not normally distributed. Table S1 lists the data transforms utilized for each measurement.

Table A. Data transform applied for each migration parameter.

**Measurement**	**Transformation**
Percent of cells migrating with/against flow direction	None
Percent of time migrating with/against flow direction	Log_{10}
Speed	Log_{10}
Displacement	Log_{10}
Confinement ratio	None
Arrest coefficient	None

Average Cell Speed (with Error Bars)

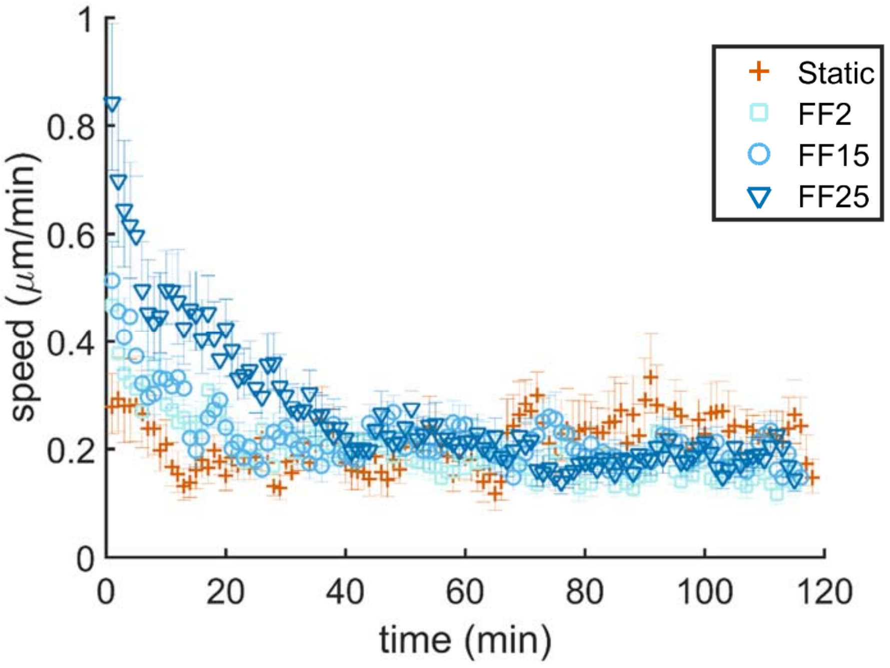

Fig. B. Average cell speed with error bars. Fig. 2a in the main text is shown here with error bars.

Effects of ROCK-shRNA on Osteoblast Migration

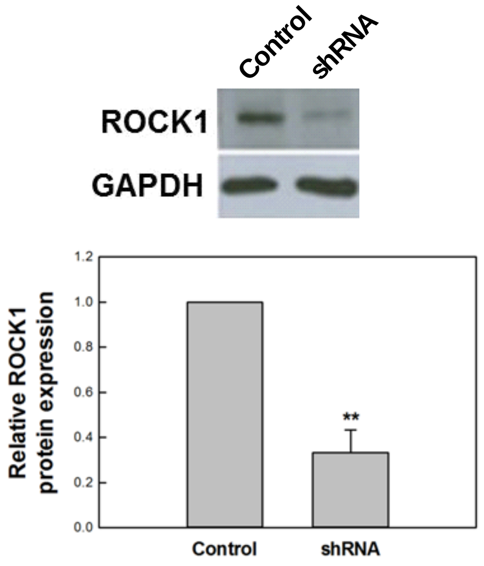

Fig. C. shRNA induces interference of ROCK1 protein expression in osteoblasts. Western immunoblotting was performed to check ROCK silencing via shRNA-ROCK1 plasmid (Santa Cruz Biotechnology, sc-36432-SH). About 67% silencing in ROCK was obtained as relative to vector control (**: $p < 0.01$, $n = 3$). GAPDH loading control is shown.

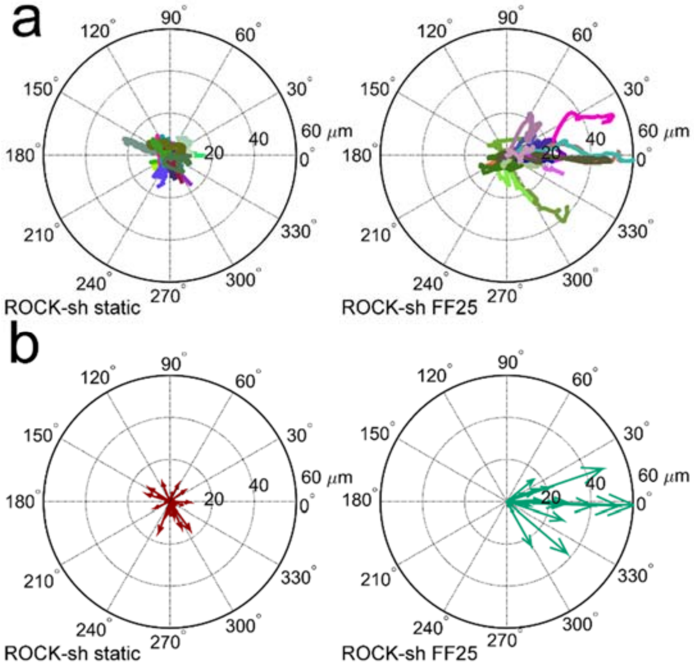

Fig. D. Cell migration tracks under ROCK-shRNA have a trend of migrating with the flow direction. (a) The raw tracks are shifted to the plot origin to show the migration trend. Cells with ROCK-shRNA under static condition migrate randomly without a biased direction. Under fluid flow (FF25), cells with ROCK-shRNA migrate from left to right following the flow direction. (b) These trends are clearer in the compass plots where an arrow connects the track start and end points.

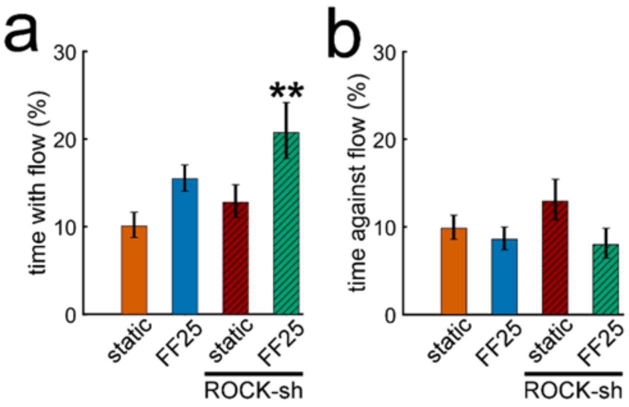

Fig. E. Cells with ROCK-shRNA under fluid shear (FF25) spent the most time migrating with the flow. (a) This difference was significant compared to the static vector control group. (b) There was no trend in time spent migrating against the flow direction. **: $p < 0.01$ compared with vector control static.

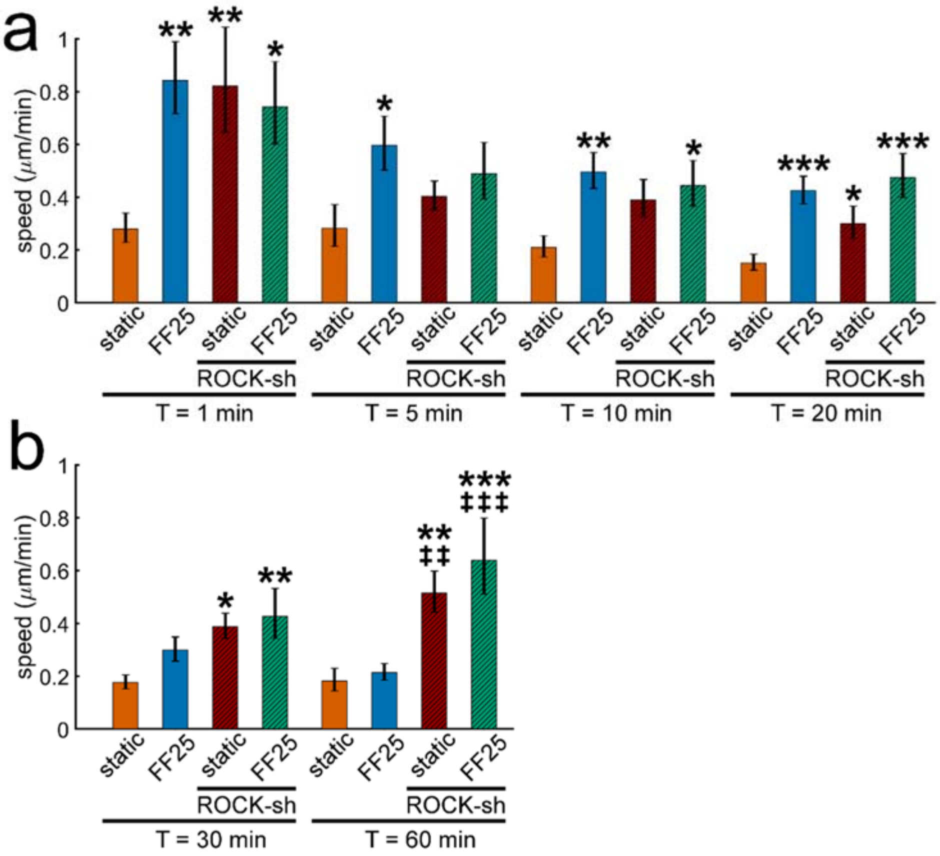

Fig. F. The ROCK-shRNA cells maintained higher cell speed even after initial period. (a) The average cell speed decreased after the initial shear onset for all conditions. (b) The long-term (e.g., 30 min and 60 min) average speeds of the ROCK-shRNA groups were significantly greater than those of vector control static and FF25 groups. *: comparison with vector control static. ‡: comparison with vector control FF25. Single, double, and triple symbols represent $p < 0.05$, 0.01, and 0.001, respectively.

Table B. Motility coefficients of osteoblasts under ROCK-shRNA.

	**shRNA-ROCK osteoblasts**					**Vector control osteoblasts**				
	**Short-term**				**Long-term**	**Short-term**				**Long-term**
	**5 min**	**10 min**	**20 min**	**30 min**	**120 min**	**5 min**	**10 min**	**20 min**	**30 min**	**120 min**
**Static**	1.442	1.017	0.719	0.6262	0.5074	1.83	3.93	3.65	2.62	1.29
**FF25**	1.873	2.091	2.03	1.951	3.069	3.43	3.20	2.59	1.95	0.80
